# Supplementary material for: Links between Neuroanatomy and Neurophysiology with Turning Performance in People with Multiple Sclerosis
Source: Sensors (Basel). 2023 Sep 3;23(17):7629. doi: 10.3390/s23177629 (PMC10490793; doi:10.3390/s23177629)
Supplement: Supplementary file 1 [file sensors-23-07629-s001.zip › sensors-2527670-supplementary.pdf]

Figure S1. Left hemisphere associations between neuroanatomical structure and turning performance and neurophysiological function and turning performance.

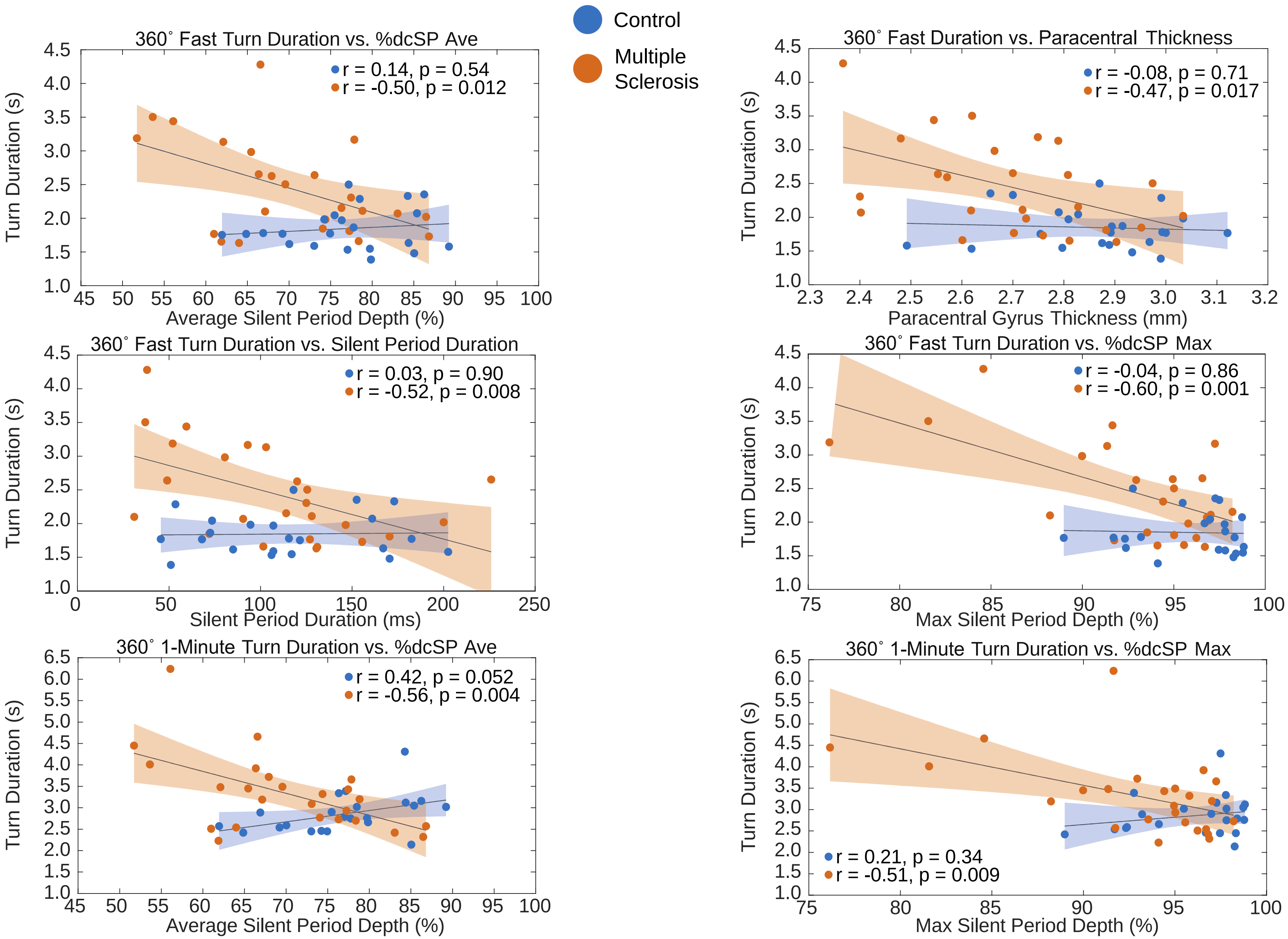

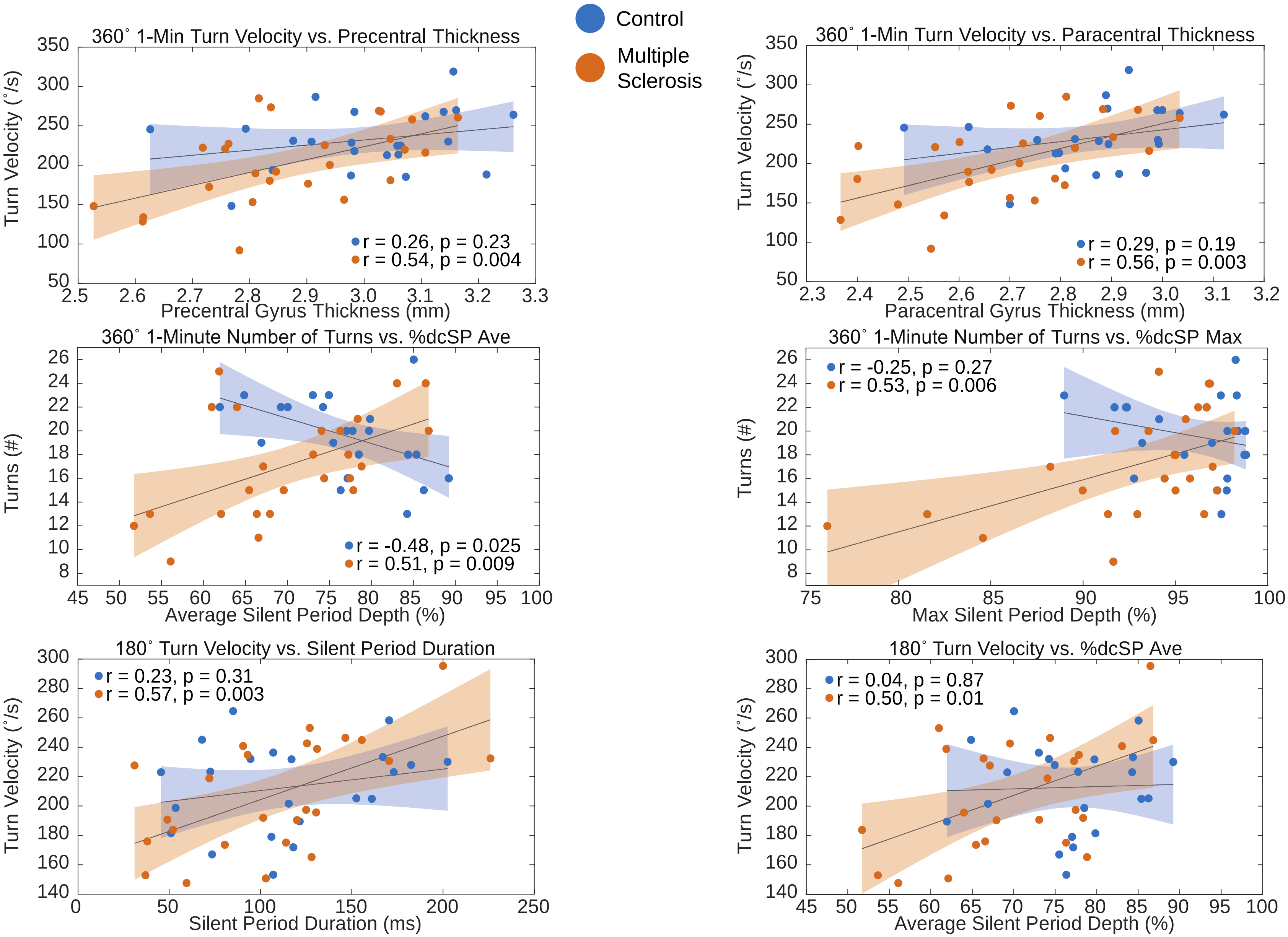

Control
Multiple Sclerosis
360˚ 1-Min Turn Velocity vs. Precentral Thickness
Turn Velocity (˚/s)
Precentral Gyrus Thickness (mm)
r = 0.26, p = 0.23
r = 0.54, p = 0.004
360˚ 1-Min Turn Velocity vs. Paracentral Thickness
Turn Velocity (˚/s)
Paracentral Gyrus Thickness (mm)
r = 0.29, p = 0.19
r = 0.56, p = 0.003
360˚ 1-Minute Number of Turns vs. %dcSP Ave
Turns (#)
Average Silent Period Depth (%)
r = -0.48, p = 0.025
r = 0.51, p = 0.009
360˚ 1-Minute Number of Turns vs. %dcSP Max
Turns (#)
Max Silent Period Depth (%)
r = -0.25, p = 0.27
r = 0.53, p = 0.006
180˚ Turn Velocity vs. Silent Period Duration
Turn Velocity (˚/s)
Silent Period Duration (ms)
r = 0.23, p = 0.31
r = 0.57, p = 0.003
180˚ Turn Velocity vs. %dcSP Ave
Turn Velocity (˚/s)
Average Silent Period Depth (%)
r = 0.04, p = 0.87
r = 0.50, p = 0.01
